# Stuttering Interneurons Generate Fast and Robust Inhibition onto Projection Neurons with Low Capacity of Short Term Modulation in Mouse Lateral Amygdala

**DOI:** 10.1371/journal.pone.0060154

**Published:** 2013-03-19

**Authors:** Chen Song, Xiao-Bin Xu, Ye He, Zhi-Peng Liu, Min Wang, Xin Zhang, Bao-Ming Li, Bing-Xing Pan

**Affiliations:** 1 Laboratory of Fear and Anxiety Disorder, Institute of Life Science, Nanchang University, Nanchang, China; 2 Department of Pharmacology, Nanchang University, Nanchang, China; Georgia Regents University, United States of America

## Abstract

The stuttering interneurons (STi) represent one minor subset of interneuron population and exhibit characteristic stuttering firing upon depolarization current injection. While it has been long held that the GABAergic inhibitory transmission largely varies with the subtype identity of presynaptic interneurons, whether such a rule also applies to STi is largely unknown. Here, by paired recording of interneuron and their neighboring projection neuron in lateral amygdala, we found that relative to the fast spiking and late spiking interneurons, the STi-evoked unitary postsynaptic currents onto the projection neurons had markedly larger amplitude, shorter onset latency and faster rising and decay kinetics. The quantal content and the number of vesicles in the readily releasable pool were also larger in synapses made by STi versus other interneurons. Moreover, the short-term plasticity, as reflected by the paired pulse depression and depolarization-induced suppression of inhibition, was the least prominent in the output synapses of STi. Thus, the fast and robust inhibition together with its low capacity of short term modulation may suggest an important role for STi in preventing the overexcitation of the projection neurons and thus gating the information traffic in amygdala.

## Introduction

The lateral nucleus of amygdala (LA), a gatekeeper of the multimodal sensory information from cortical and subcortical areas entering the amygdala, has been generally recognized to play a critical role in the acquisition, storage and expression of emotional information such as fear and anxiety [1]–[3]. Whereas the excitatory projection neurons (PNs) mediate the signal transfer between LA and its down- and upstream brain areas [4]–[6], the local GABAergic interneurons (INs) prevent the overexcitation of PNs and ensure the appropriate expression of fear and anxiety through establishing the highly inhibitory tone in amygdala [7], [8]. The impairment in amygdala inhibition closely correlates with the development of a series of mental disorders such as posttraumatic stress disorders [9], [10].

As in hippocampus and cortex, the INs in LA exhibit wide diversity in terms of their morphological, neurochemical and electrophysiological features [11]. Studies using immunostaining of their molecular markers have revealed some major non-overlapping subtypes of INs in LA with each expressing parvalbumin (PV), cholecystokinin (CCK) or somatostatin (SOM) [12]–[14]. Based on their spiking response to the current step injection, the INs can be classified into multiple sets such as fast spiking interneurons (FSi), late spiking interneurons (LSi), accommodating INs (ACi) and STi [15]–[18]. Of these, the STi constitute a minor subset of the IN population and are characterized by bursts of action potentials intermingled with variable quiescent periods upon the sustained depolarization current injection.

Accumulating evidence has shown that the inhibition imposed on the target neurons largely depend on the subtype identity of the presynaptic Ins [19], [20]. For example, in hippocampus, the fast-spiking basket cells generate fast and strong perisomatic inhibition onto the PNs, the late-spiking neurogliaform cells, on the other hand, provide slow and weak inhibitory signal through the connections distal to the soma [21]. Although the STi have been identified in multiple brain areas such as cortex, striatum and amygdale [15], [16], [22], [23], very little is known about the properties of the unitary inhibitory transmission mediated by STi. It is yet unclear whether the rule also applies to the STi that presynaptic INs dictate the inhibitory transmission. Specifically, do the STi, which fire in a pattern clearly distinguishable from other INs, also generate unique form of inhibition onto their nearby PNs? To answer this, we made simultaneous recording of IN-PN pairs from GAD-67 GFP knock-in mice and compared the properties of unitary inhibitory postsynaptic currents (uIPSCs) in connections made by STi and other IN subtypes. We found that relative to other INs, the STi evoked faster and more robust inhibition onto nearby PNs. Moreover, the short-term plasticity was less prominent in the output synapses of STi.

## Materials and Methods

### Slice preparation

All experimental procedures involving animals were approved by the Animal Ethics Committee of Nanchang University. Amygdala slices were prepared as previously described from 4–5 weeks old heterozygous GAD67-GFP(Δneo) male mice in which GFP is selectively expressed in Ins [24], [25]. Briefly, mice were sacrificed by decapitation and brains were quickly removed to ice-cold oxygenated (95% O_2_/5% CO_2_) artificial cerebrospinal fluid (ACSF) containing (in mM): 124 NaCl, 2.5 KCl, 1 MgSO_4_, 2.5 CaCl_2_, 10 glucose, and 26 NaHCO_3_ (pH 7.30). Slices containing LA of about 350 µm were cut with a Leica VT 1000S tissue slicer and maintained at room-temperature for at least one hour before recording.

### Electrophysiological Recording

Slices were transferred to a recording chamber continuously superfused with ACSF at a constant rate of about 60 ml/h and the recording temperature was held at 29±1°C. Dual whole cell recordings were performed in IN-PN pairs in LA with an EPC-10 amplifier and Patchmaster software (HEKA Elektronik, Germany). The PNs were visualized under guidance of DIC/infrared optics and the INs by their green fluorescence. Data were filtered at 2 K Hz using the patch-clamp amplifier circuitry and digitized at 10 k Hz. The patch pipettes for recording PNs were filled with (in mM): 100 CsCl, 20 Cs-methanesulfonate, 5 NaCl, 2 MgCl_2_, 10 HEPES, and 0.2 EGTA, 2 ATP-Na, 0.1 GTP-Na. The CsCl and Cs-methanesulfonate were replaced by the same concentration of Kgluconate in pipettes for recordings of INs. The pH was adjusted to 7.3 with CsOH or KOH and osmolarity to 285Osm with sucrose. The membrane potentials were corrected by a junction potential of about 12 mV. Series resistance (Rs) was in the range of 10–20 MΩ and monitored throughout experiments. If Rs changed more than 20% during recording, the data were not included in analysis.

To examine the uIPSCs in IN-PN synapses, a short term current injection (5 ms, 1nA) was delivered to evoke single action potential in INs and the postsynaptic response in PNs was monitored. To induce DSI, single action potentials in INs were evoked at 0.2 Hz and the basal uIPSCs (10–12 sweeps) in PNs were recorded, followed by a depolarizing pulse from −70 to 0 mV applied to the PNs for 5 seconds. Three seconds later, subsequent IPSCs were continuously collected at 0.2 Hz. The magnitude of suppression was calculated as the percentage of reduction in average amplitudes of 5–7 consecutive uIPSCs after depolarization relative to the mean amplitude of basal uIPSCs. To examine the quantal properties in IN-PN connections, 30 short current pulses (2 ms, 1nA) were delivered to the INs at high frequency (200 Hz) and the uIPSCs in their paired PNs were recorded.

### Data Analysis and Statistics

The uIPSCs parameters were measured and averaged from individual responses (n≥15). The onset latency was determined by the time interval between the peak of presynaptic spike and the onset of uIPSCs. The decay time constant was measured by fitting the falling phases of individual uIPSCs with a monoexponential function. uIPSCs superimposed by the spontaneous IPSCs or with the mean amplitude less than 15 pA were excluded for the analysis. Such criterion excluded 3 FSi-PN and 3 LSi-PN pairs which were synaptically connected. The electrophysiological parameters of the action potential were measured as previously described [26].

One way ANOVA and unpaired *t* test were used appropriately to compare the means of uIPSCs parameters, paired pulse ratio (PPR) and DSI. The interrelations between the uIPSCs parameters were derived from the *r* value of the fitted linear regression and tested by Person's test. Principal component analysis (PCA) was applied to the 5 uIPSCs parameters including the amplitude, charge transfer, onset latency, 10–90% rise time and decay time constant. Two components with eigenvalues greater than 1 were extracted with correlation matrix analysis and accounted for 89.4% of the total variability. Component scores were calculated using a regression method implemented in SPSS 10 (SPSS Inc., Chicago, IL).

## Results

**Figure 1:**
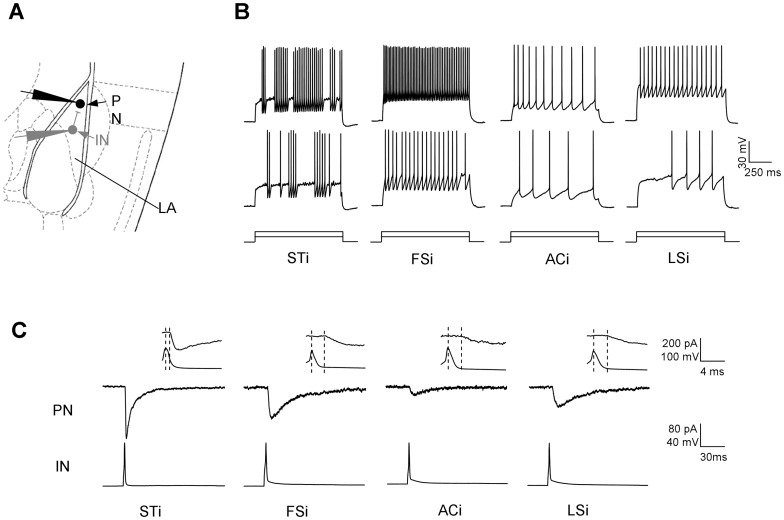
The uIPSCs evoked by different subtypes of INs onto PNs in LA. **A**, Schematic graph showing the simultaneous recording of IN-PN pairs in LA. **B**, Representative traces showing the firing patterns of STi, FSi, LSi and ACi upon injection of 1 s threshold current (middle trace) and current 80pA above the threshold (top trace). The bottom shows the pattern of current injection onto INs. **C,** Representative traces showing the uIPSCs in individual PNs evoked by distinct subtypes of INs. Insets show the expanded graph and the dashed lines indicate the peak of spike in INs (left) and the onset of uIPSCs in PNs (right).

We have made stable recordings from a total of 168 IN-PN pairs in LA (Fig. 1A). In these pairs, 93 INs were found to have functional inhibitory connections onto the PNs. Based on their spiking patterns upon the sustained depolarization current injection, the INs were identified as STi, FSi, LSi and ACi respectively. As shown in Fig. 1B, the STi discharge with spike bursts separated by unpredictable quiescent periods upon suprathreshold current injection. The FSi show fast non-adaptive firing throughout the current injection. By contrast, the firing of ACi exhibit clear adaptation upon the current injection. While exhibiting a similar non-adaptive firing pattern as FSi upon strong current injection, the LSi display delayed spikes preceded by a ramp-like depolarization in response to the threshold current injection. The probabilities for the INs to make synaptic connection with nearby PNs were 0.53 for STi (18/34), 0.62 for FSi (32/52), 0.20 for ACi (3/15) and 0.60 for LSi (40/67). The examples of the uIPSCs in synapses made by distinct INs were shown in Fig. 1C. Since only 3 ACi were found to have synaptic connection with the paired PNs, we suspended further comparison of the ACi-PN synapses with those by other INs. In all the synaptically connected pairs, the uIPSCs in PNs were precisely time-locked to the presynaptic spike in INs and completely blocked by addition of 10 µM bicucculine, a GABAaR antagonist, indicating a monosynaptic GABAergic locus for the uIPSCs in PNs (data not shown). We found that the vast majority of the presynaptic spikes in INs readily evoked postsynaptic uIPSCs in PN. The failure events were very occasionally observed in FSi- (1.06±0.61%) and LSi-PN connections (1.08±0.53%) with no failure being detected in the STi-PN connections, yielding an extremely low failure rate (0.86±0.32%) for the whole trials. The highly reliable GABAergic transmission might constitute one important aspect of the physiological basis for the highly inhibitory tone in amygdale [27].

**Figure 2:**
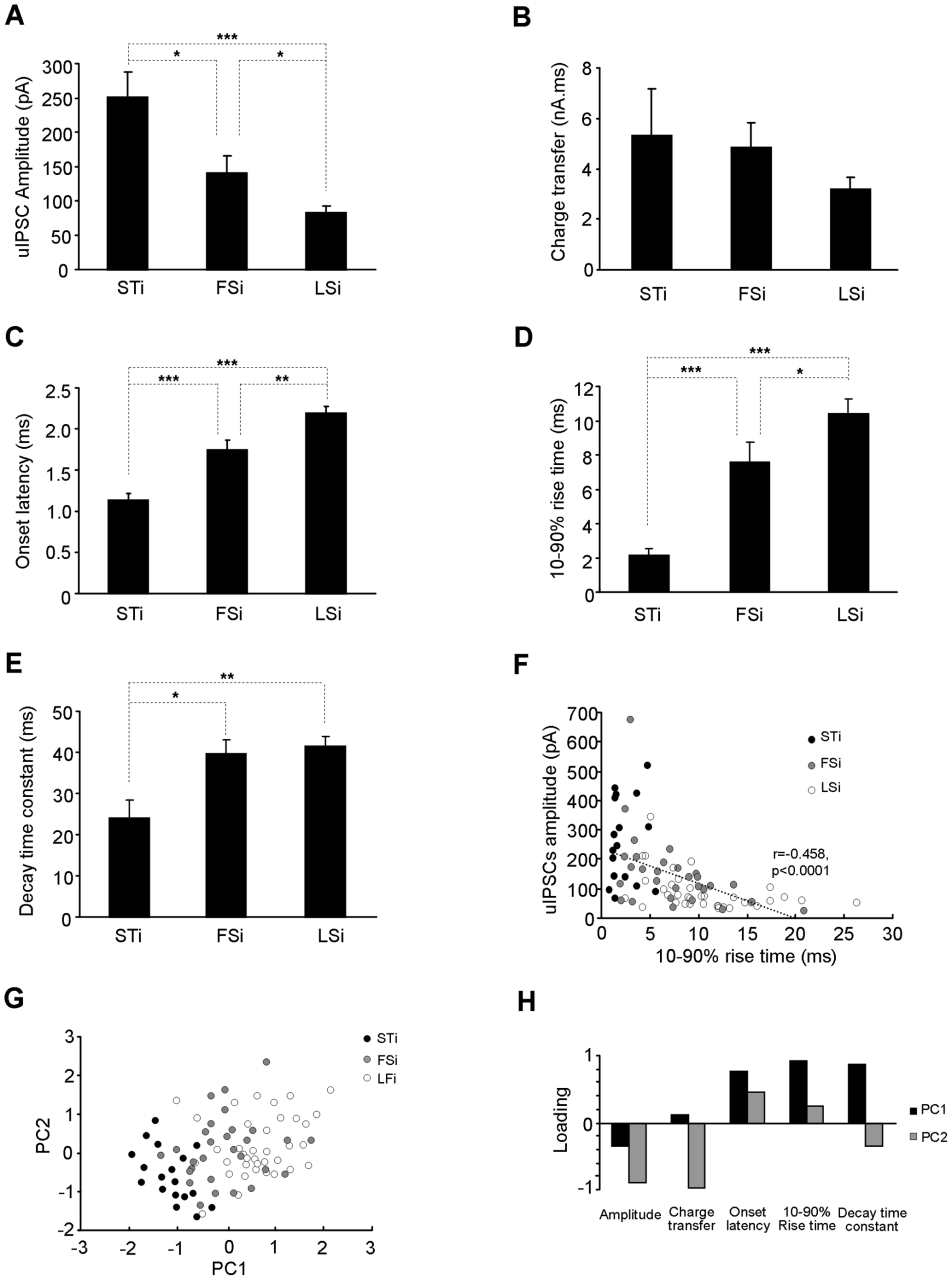
The STi-evoked uIPSCs onto PNs have higher amplitude and shorter time course. **A–E**, Comparisons of the amplitude (A), charge transfer (B), onset latency (C), 10–90% rise time (D) and decay time constant (E) of the uIPSCs in synapses made by STi, FSi and LSi onto PNs. *p<0.05, **p<0.01, ***p<0.0001. **F**, Strong correlation between the amplitude and 10–90% rise time of the whole uIPSCs. The data points from synapses made by distinct INs were indicated. **G**, Scatterplot of the parameters of uIPSCs by 3 subtypes of INs in the principal component plane. Each principal component is a linear combination of 5 parameters shown in A-E. PC1 strongly correlates with parameters reflecting the time course and PC2 with those representing the strength of uIPSCs. **H**, Principal component loadings (correlations) for each parameter are shown.

The comparison of the uIPSCs properties revealed some striking features for those in connections formed by STi. The amplitude of uIPSCs in STi-PN synapses ranged from 69.4 pA to 519.5 pA with a mean value of 251.5±37.8 pA (n = 18), which was significantly higher than the value of 140.4±26.6 pA (n = 29, p = 0.035) and 82.3±10.6 pA (n = 37, p<0.001) for synapses made by FSi and LSi respectively (Fig. 2A). The charge transfer of the uIPSCs, on the other hand, was only slightly but insignificantly larger in the output synapses of STi (STi: 5.31±1.87 nA.ms; FSi: 4.86±0.96 nA.ms; LSi: 3.20±0.48 nA.ms; Fig. 2B). We also found considerable differences in the kinetics of uIPSCs in connections made by these 3 clusters of INs. The onset latency of uIPSCs was the shortest in those formed by STi (STi: 1.13±0.09 ms; FSi: 1.78±0.10 ms; LSi: 2.20±0.07 ms, p<0.001, Fig. 2C). The 10–90% rise time of uIPSCs in STi-PN synapses was only about 20% of that in those made by LSi and 30% by FSi (STi: 2.12±0.38 ms; FSi: 7.45±1.20 ms; LSi: 10.30±0.88 ms, p<0.001, Fig. 2D). Moreover, their decay time constant was also markedly shorter (STi: 24.11±4.32 ms; FSi: 39.66±3.48 ms; LSi: 41.43±2.48 ms, p = 0.006, Fig. 2E). Thus, relative to the FSi and LSi, the STi evoked uIPSCs with larger amplitude and shorter kinetics on the neighboring PNs in LA. To examine whether the uIPSCs varying with the presynaptic IN subtypes also existed in other subregions of amygdala, we extended the above comparisons to the basoamygdala and found that the STi-evoked uIPSCs also differed markedly from those by other INs ( Fig. S1). Thus, the pattern that the uIPSCs varies with the IN subtypes appears similar in lateral and basal nucleus of amgydala.

Further analysis of the correlations among the uIPSC parameters revealed strong negative correlation between the amplitude and rise time of the whole population of uIPSCs (r = −0.458, p<0.001, Fig. 2F), implying that the uIPSCs of larger amplitude in the output synapses of STi might be less affected by the electronic filtering. In support of this, the above correlation seemed to emerge in uIPSCs evoked by FSi and LSi but not STi (STi: r = 0.176, p = 0.621; FSi: r = −0.489, p = 0.018, LSi: r = −0.445, p = 0.014). Since it was widely demonstrated that the electronic filtering did not significantly affect the inhibitory currents in synapses close to the soma [28], [29], the lack of correlation between the uIPSCs amplitude and rise time in STi-PN synapses suggest that STi most likely innervate the PNs proximal to their soma. The significant correlations were also observed within the parameters representing either the strength or kinetics of uIPSCs (amplitude versus charge transfer: r = 0.757, p<0.001; onset latency versus 10–90% rise time: r = 0.801, p<0.001; onset latency versus decay time constant, r = 0.398, p = 0.001; 10–90% rise time versus decay time constant: r = 0.669, p<0.001), making it somewhat redundant to use these intrinsically related parameters to denote the uIPSCs evoked by distinct INs. To reduce the redundancy, we performed PCA on the 5 variables reflecting the uIPSCs. The first two principal components (PC1 and PC2) accounted for 89.37% variability of the total variance (eigen values: PC1, 2.623; PC2: 1.788; cumulative percentage: PC1, 53.95; PC2, 89.37) and were plotted against each other (Fig. 2G and H). The PC1 strongly correlated with the kinetics and PC2 with the strength of uIPSCs. In the PC1-PC2 plane, most of the uIPSCs evoked by STi were segregated from those by FSi and LSi. The uIPSCs in FSi-PN synapses composed the intermediate portion and exhibited clear overlap with those in the LSi-PN synapses. Comparisons of the PC1 and PC2 values revealed clear between-group differences in both PC1 and PC2 (PC1, STi: −1.26±0.14; FS: −0.11±0.19; LSi: 0.50±0.12, p<0.001; PC2, STi: −0.54±0.21; FS: 0.12±0.19; LS: 0.03±0.12, p = 0.033). Thus, the uIPSCs differed in both their kinetics and strength in the synapses made by STi and other INs.

**Figure 3:**
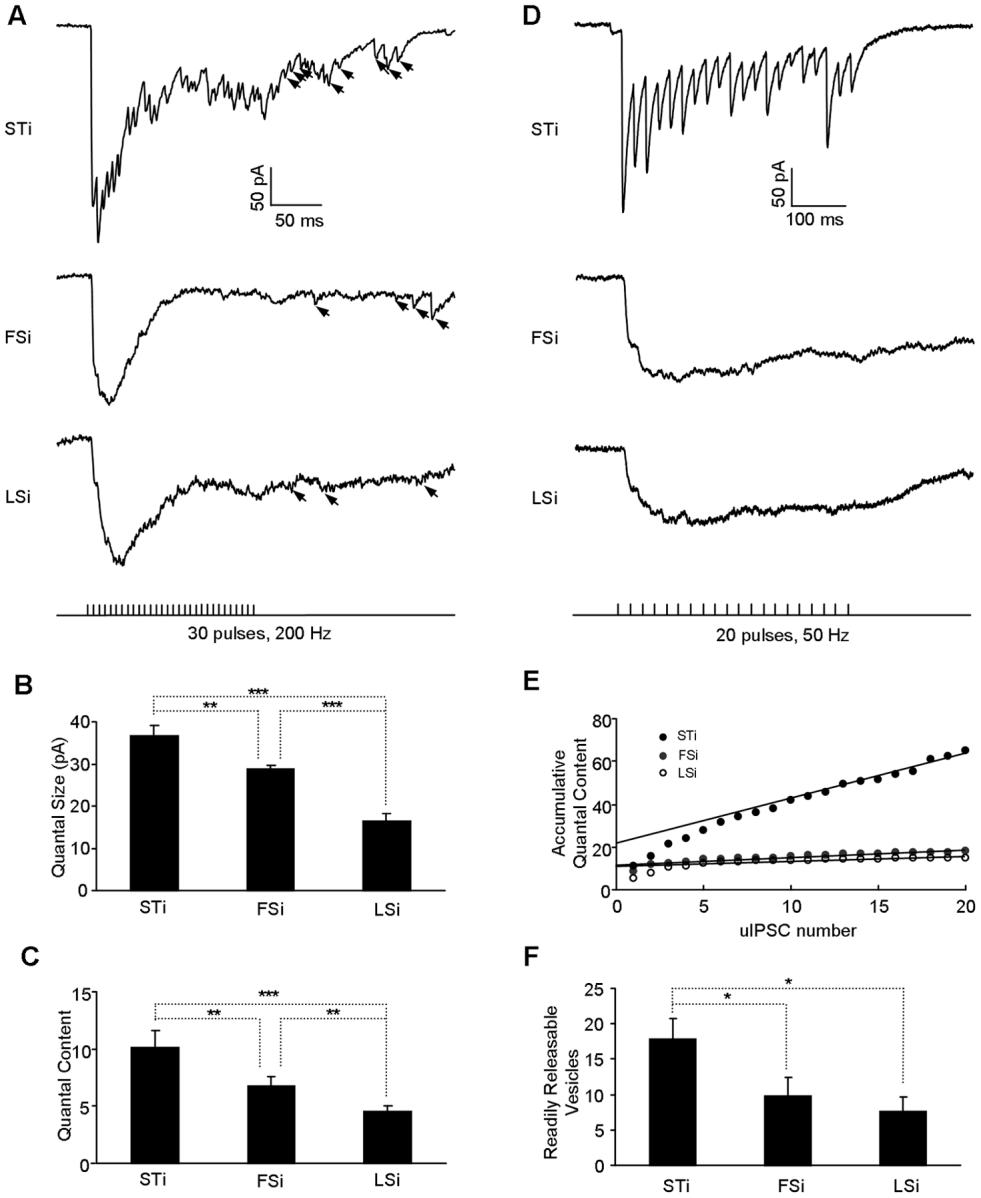
The quantal properties of inhibitory synapses made by distinct INs. **A,** Representative traces showing the responses of postsynaptic PNs to 30 current pulses delivered at 200 Hz to STi (top), FSi (middle) and LSi (bottom). The arrows indicate the uIPSCs resulting from asynchronous quantal release after the cessation of presynaptic stimuli. **B–C,** Comparisons of the quantal size (B) and quantal content (C) in connections made by STi, FSi and LSi. **D,** Representative traces showing the responses of postsynaptic PNs to 20 current pulses delivered at 50 Hz to STi (top), FSi (middle) and LSi (bottom). Each trace is averaged from 3–4 consecutive recordings in the same inhibitory synapses. **E,** Plot of the accumulative quantal content in D as a function of uIPSCs number. To determine the accumulative quantal content, a linear regression line was fitted to the last 8 events which represented the steady-state component of the train. Back-extrapolation to the start of the train was used to estimate the number of quanta released before pool replenishment. For examples shown in D, 22, 13 and 13 quanta were estimated for synapses made by STi, FSi and LSi respectively. **F,** Comparison of the readily releasable vesicles in synapses by distinct INs. *p<0.01; **p<0.01; ***p<0.001.

To investigate the synaptic mechanisms underlying the uIPSCs differences described above, we attempted to compare the quantal properties in IN-PN synapses. To estimate the quantal size in synapses made by different INs, thirty current pulses were delivered to the INs at 200 Hz and the postsynaptic IPSCs in PNs were recorded. As shown in Fig. 3A, numerous small IPSCs emerged after the cessation of APs which were shown to mostly stem from asynchronous release of single quanta [30]. Comparisons of these small IPSCs revealed that the quantal size was much larger in the connections made by STi versus FSi and LSi (STi: 36.8±2.4 pA, n = 4; FSi: 28.8±0.92 pA, n = 6; LSi: 16.4±1.8pA, n = 6, p<0.001, Fig. 3B). The quantal content, as calculated by dividing the amplitude of 1^st^ uIPCS by the quantal size, was also larger in the output synapses of STi (STi: 10.2±1.4; FSi: 6.8±0.8; LSi: 4.5±0.5, p = 0.008, Fig. 3C). Thus, compared to those made by FSi and LSi, the STi-PN synapses had larger quantal size and the firing of STi would lead to the release of more vesicles, thus causing a more robust uIPSCs in the postsynaptic PNs. We further analyzed the accumulated uIPSCs upon trains of APs at 50 Hz and found that the number of the vesicles in the readily releasable pool was also larger in the terminals of STi relative to FSi and LSi (STi: 17.8±2.9, n = 4, FSi: 9.6±2.6, n = 5, LSi: 7.6±2.1, n = 5, p = 0.023, Fig. 3D–F). Altogether, the above findings highlighted considerable differences in the quantal properties in synapses established by different INs. Since the APs in individual neurons also impact the vesicle release in their terminals, we next compared the parameters of the APs in different INs. As shown in Table 1, there were conspicuous differences in both the amplitude and kinetics of the APs between STi and LSi. By contrast, for STi and FSi, their APs were very similar.

**Table 1:** Comparisons of the action potential properties in distinct INs.

IN	AP threshold (mV)	AP amplitude (mV)	AP half-width (ms)	10–90% rise time (ms)	90–10% decay time (ms)	fAHP (mV)
STi	−45.36±0.83***	57.69±1.11**	0.71±0.03***	0.51±0.03**	0.73±0.04***	18.75±0.74**
FSi	−47.98±0.66***	58.33±1.34**	0.72±0.03***	0.44±0.03***	0.76±0.04***	18.95±0.84**
LSi	−39.24±1.44	51.81±1.24	1.08±0.08	0.66±0.06	1.24±0.08	22.95±0.67

AP: action potential; fAHP: fast afterhyperpolarization. **p<0.01 *vs* LSi; **p<0.001 *vs* LSi.

**Figure 4:**
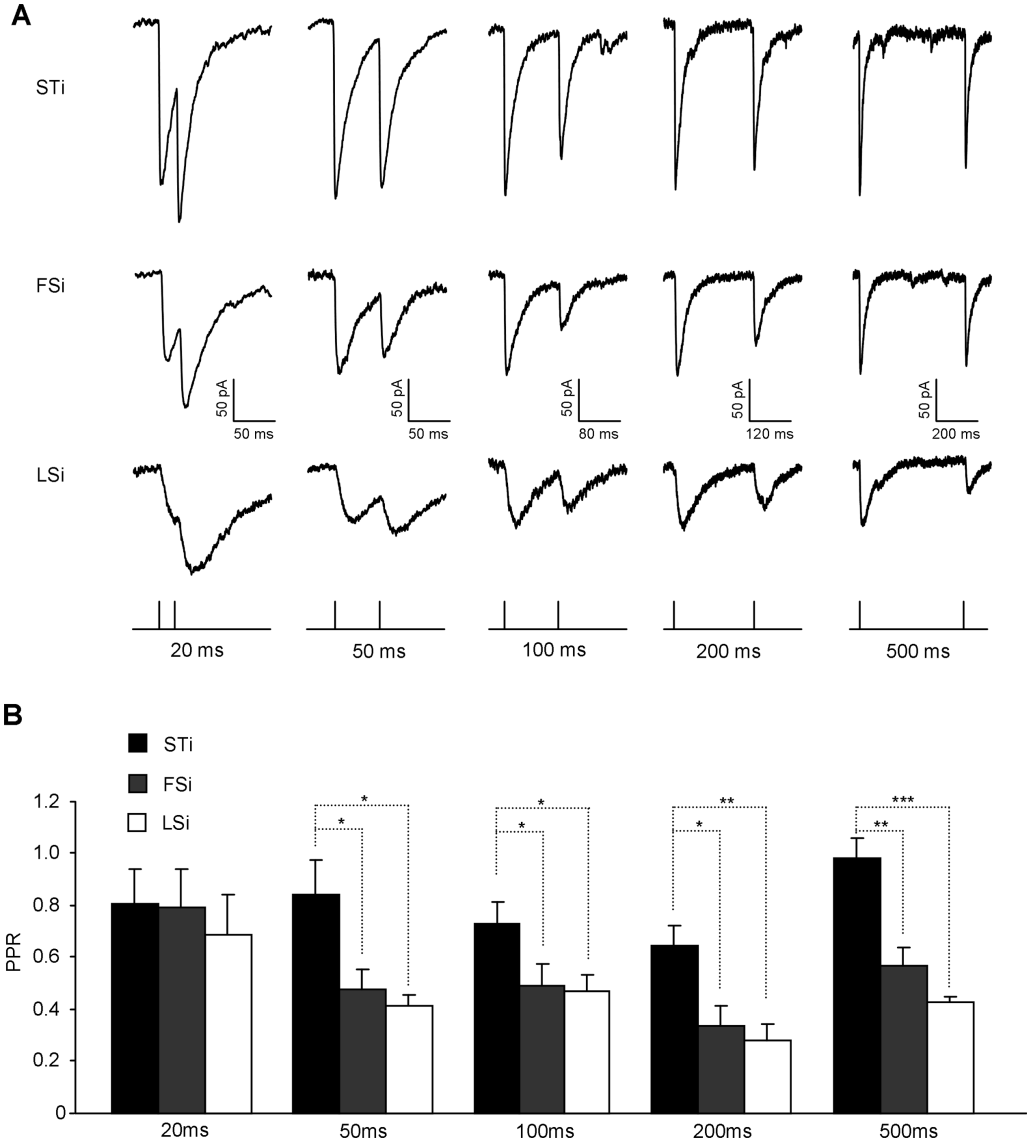
Weaker PPD in connections made by STi versus FSi and LSi. **A**, Representative traces showing the uIPSCs in PNs evoked upon two successive action potentials in their paired INs (top: STi, middle: FSi and LSi) separated by intervals varying from 20 ms to 500 ms. The bottom showing the pattern of current injection delivered to INs at different intervals. **B**, The summary plots of paired pulse ratio (PPR) against the intervals in different IN subtypes. *p<0.05; **p<0.01; ***p<0.001.

**Figure 5:**
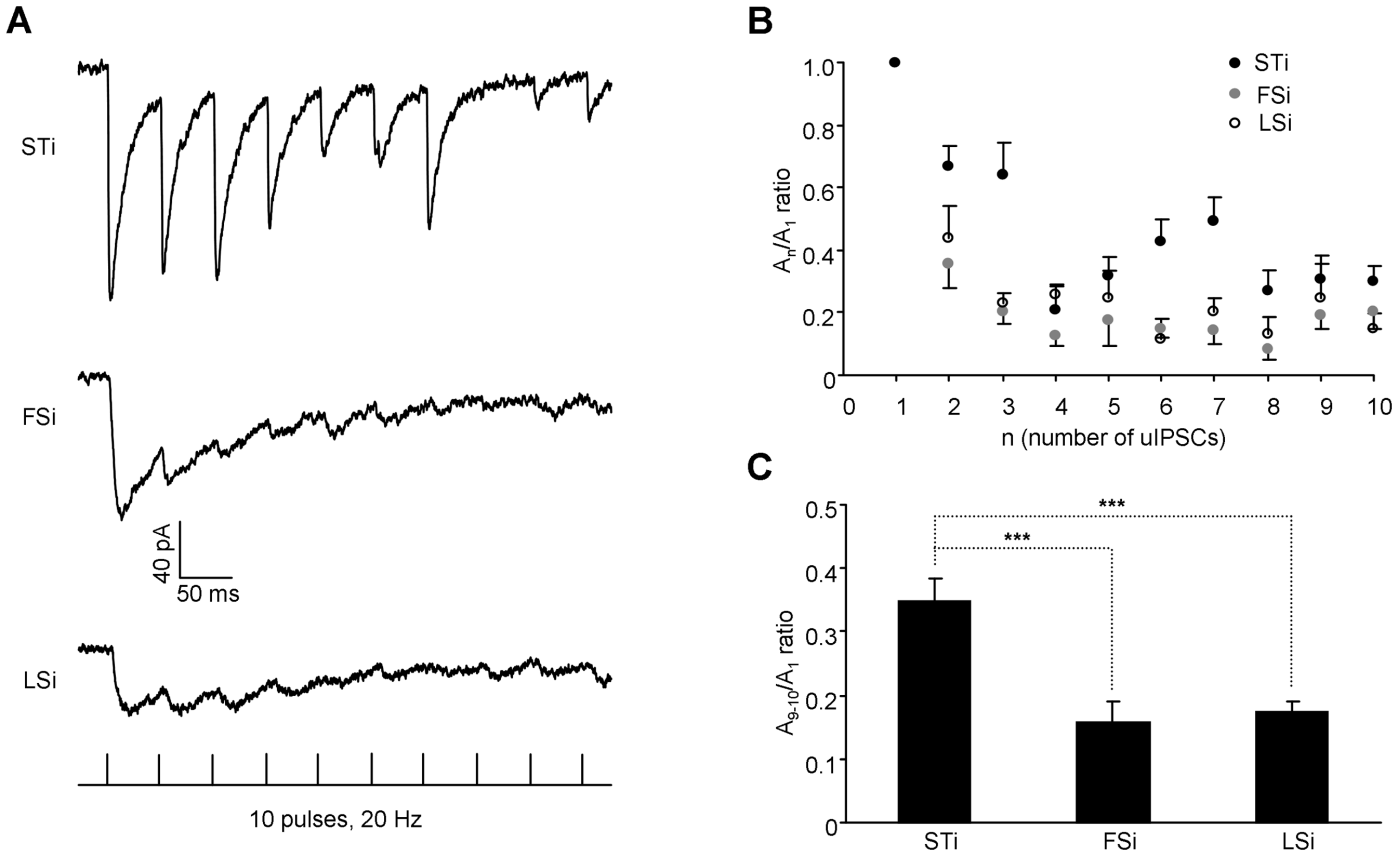
Decay of the uIPSCs in PNs upon trains of action potentials in INs. **A,** Representative traces showing the uIPSCs in PNs in response to 10 current pulses delivered at 20 Hz to their paired INs (Top: STi; Middle: FSi and LSi). The bottom showing the pattern of current pulses delivered to INs. **B,** Plots of *A_n_*/*A_1_* ratio against the number of uIPSCs, where *A_n_* refers to the amplitude of the consecutive uIPSCs of the train and *A_1_* to the amplitude of the 1^st^ uIPSC. Note that the uIPSCs decayed more slowly in STi-PN synapses. **C,** Comparison of the ratios between *A_9–10_* (the average amplitude of the 9^th^ and 10^th^ uIPSC) and *A_1_* in different IN-PN pairs. ***p<0.001.

Besides the basal transmission, the short-term plasticity in the inhibitory synapses was also shown to depend on the cell type of presynaptic INs [19], [31]. To test whether this also held true for STi, we explored the short-term modification of uIPSCs in connections formed by STi, FSi and LSi. We first compared the postsynaptic uIPSCs in PNs upon two successive APs in INs separated at intervals varying from 20 ms to 500 ms. Although the paired pulse ratio (PPR) appeared similar among different pairs at the interval of 20 ms (STi: 0.82±0.14, n = 4; FSi: 0.79±0.16, n = 5; LSi: 0.68±0.16, n = 6, p = 0.834, Fig. 4), it was conspicuously larger in synapses established by STi when the interval was set at 50 (STi: 0.84±0.13; FSi: 0.48±0.08; LSi: 0.41±0.04, p = 0.032), 100 (STi: 0.73±0.10; FSi: 0.50±0.09; LSi: 0.47±0.07, p = 0.046), 200 (STi: 0.64±0.08; FSi: 0.36±0.07; LSi: 0.28±0.07, p = 0.011) or 500 ms (STi: 0.97±0.09; FSi: 0.57±0.08; LSi: 0.42±0.02, p = 0.004). Moreover, upon trains of APs in INs, the uIPSCs decayed more slowly in the output synapses of STi, as suggested by the higher ratio between the amplitude of the 1^st^ uIPSCs with the average amplitude of the 9^th^ and 10^th^ uIPSCs in these synapses. (STi: 0.34±0.04, n = 4; FSi: 0.16±0.03, n = 5; LSi: 0.17±0.02, n = 5, p<0.001, Fig. 5).

**Figure 6:**
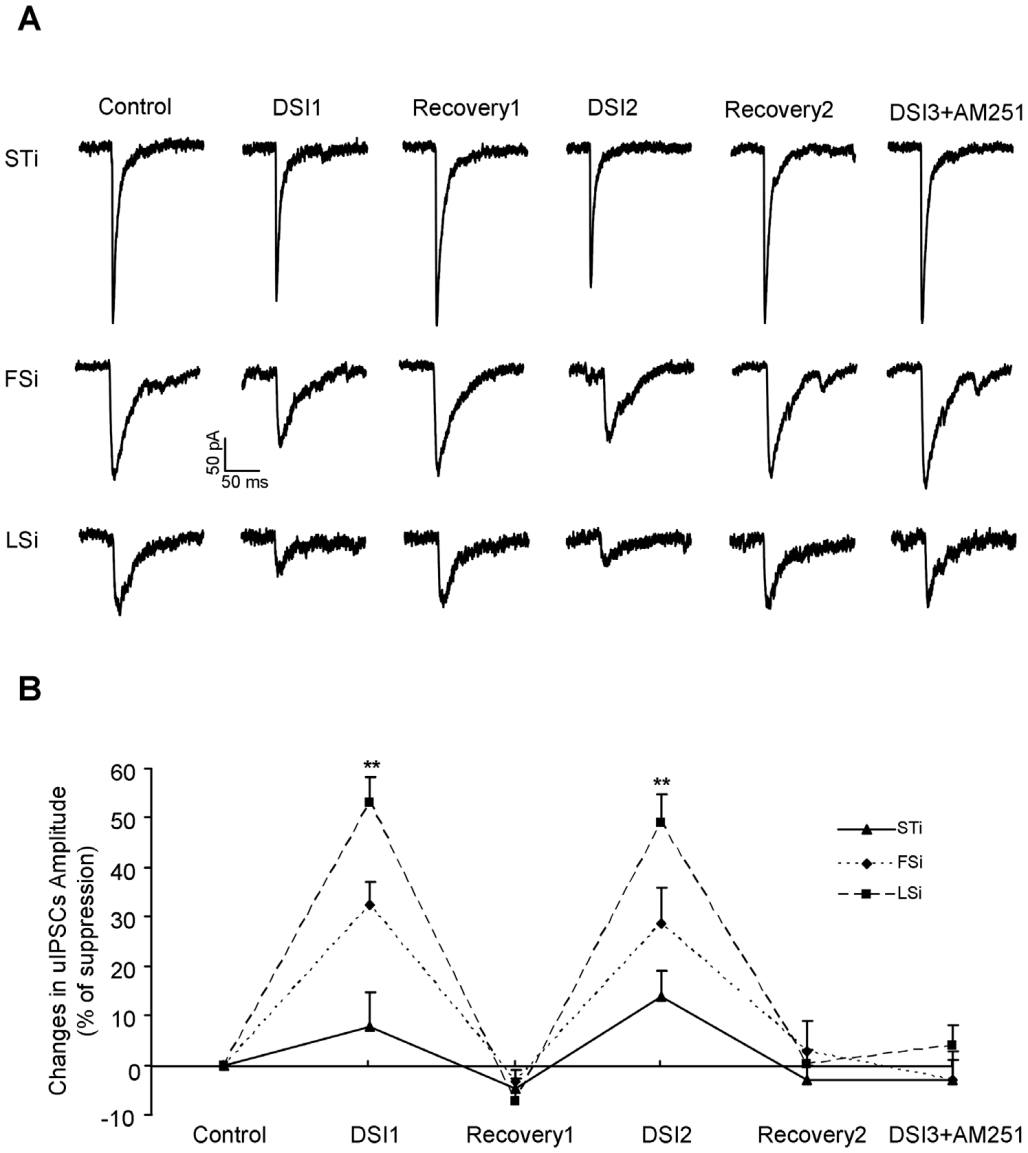
Dependence of DSI on the presynaptic IN subtypes. **A**, Representative traces of uIPSCs evoked by STi (top), FSi (middle) and LSi (bottom) at different periods of the DSI experiment. **B,** Plots of the suppression of uIPSCs amplitude at different stages of the experiment. **p<0.01 between uIPSCs evoked by STi versus FSi and STi.

We next examined whether the depolarization induced suppression of inhibition (DSI), another form of short term plasticity depending on the retrograde endocannabinoid signaling, also varied with the subtype identity of presynaptic INs in LA. Five seconds depolarization in PNs only slightly affected the uIPSCs evoked by STi. By contrast, it evoked DSI in synapses made by both FSi and LSi. Furthermore, the DSI appeared more robust in synapses made by LSi (% of suppression, STi: 10.2±7.1, n = 5; FSi: 35.3±4.7, n = 9; LSi: 53.0±5.5, n = 12; STi versus FSi and LSi, p = 0.003; FSi *vs* LSi, p = 0.033, Fig. 6A and B), implying the DSI is largely dependent on the presynaptic cell type. To confirm this, we repeated the experiment in the identical pairs after 5 minutes recovery and readily revealed similar findings (% of suppression, STi: 13.8±5.1; FSi: 32.1±7.3; LSi: 48.9±5.9; STi versus FSi and LSi, p = 0.007; FSi versus LSi, p = 0.047). In further experiments when 10 µM AM251, a cannabinoid receptor 1 (CB1) antagonist was included in the solution, the DSI in both FSi- and LSi-PN synapses was abolished (% of suppression, STi: −3.1±6.3; FSi: 4.2±4.0; LSi: −4.5±4.3; p = 0.587), indicating a critical role of CB1 activation in DSI. Collectively, our above results suggested that the uIPSCs were less capable of short term regulation in connections formed by STi versus FSi and LSi.

## Discussion

Here, we observed that, besides the characteristic stuttering firing pattern upon the depolarization current injection, the STi differed from other INs in both the unitary transmission and short term plasticity in their output synapses. Among the 3 clusters of uIPSCs generated by STi, FSi and LSi, the STi-evoked uIPSCs had the largest amplitude and fastest kinetics. The short term plasticity, on the other hand, was the least prominent in STi-PN synapses. Whereas it has been well established that the cell type of presynaptic INs largely determines the inhibitory transmission onto their target [30]–[33], we here provided evidence to extend this principle to the STi, a minor IN subset which were seldom investigated.

We first found that the STi could be readily distinguished from other INs in both the amplitude and kinetics of the uIPSCs they evoked on the PNs. In line with this, the quantal properties also differed in synapses made by STi versus other INs. Both the quantal size and the quantal content appeared the largest in these synapses. Although clear differences were observed in the APs between STi and LSi, they were very similar between STi and FSi. Thus, the AP, if yes, may only partially mediate the differences seen in the uIPSCs. Our finding that different subtypes of INs imposed different uIPSCs onto PNs, however, was at odds with a recent observation that the uIPSCs appeared indistinguishable in synapses formed by STi and FSi in PV+ IN network in basal amygdale [15]. Although the exact reason for such a discrepancy is not yet known, the different FSi involved in the current and previous studies may explain this. While being a major subtype of the PV+ INs, the FSi also composed a substantial portion of PV-/SOM+ INs which preferentially innervate the distal dendritic domain of the target cells [34], [35]. Since the INs visualized in the GAD-67 GFP knock-in mice covered nearly the whole IN population with half of them being positive for PV [25], a large fraction of FSi identified in the present work should be PV- and made innervations distal to the soma of PNs. Thus, compared with those by PV+ FSi, the uIPSCs evoked by the PV- ones would be more affected by dendritic cable filtering, causing an overall reduction in the amplitude of the FSi-evoked uIPSCs in the current study and a clear delay in their kinetics. Some of the neuronal properties were shown to be largely heterogeneous in the lateral and basal amygdale [36], [37], however, the pattern that uIPSC in postsynaptic PNs varies with presynaptic IN appeared similar in both areas.

We also observed that relative to other IN subtypes, the short term modulation was far weaker in the output synapses of STi. Multiple factors such as the number of the vesicles in RRP, inactivation of release sites and reduction of calcium influx have been proposed to modulate the short term depression in synapses [38], [39]. We propose that more vesicles in the RRP may underlie the weak PPD in STi-PN synapses. The output synapses of STi were shown to have relatively larger quantal content. Somewhat surprisingly, at most of the PPD experiments, the 2^nd^ uIPSCs also appeared larger as indicated by the higher PPR. Moreover, in these synapses, the uIPSCs decayed more slowly upon trains of APs in INs. These observations, therefore, strongly suggested that, relative to FSi and LSi, the vesicles in the RRP of STi-PN synapses were of bigger size and might undergo slower depletion upon repetitive neuronal firing. As expected, we found that the RRP size did vary with the IN subtypes and was the largest in connections made by STi. Whether other factors such as changes in calcium influx or release sites contribute to the different PPD remains unclear. In line with the weak PPD in STi-PN synapses, the presynaptic CR1-dependent DSI was nearly negligible in these synapses. In LA, the CR1 was preferentially expressed in INs expressing CCK [36], [40] and the lack of DSI in connections by STi suggested that STi were mostly negative for CCK. Consistently, none of the STi were found in the basolateral amygdala in mice with letivirus-expressing GFP under the control of the CCK promoter [26]. In many brain areas such as hippocampus and cerebral cortex, the DSI in the output synapses of FSi was reported to be absent [41], [42]. However, in the striatum, it was shown that DSI was apparent in the connections made by FSi onto the medial spiny neurons [43]. Together with the current observation of a moderate DSI in FSi-PN synapses in LA, it is likely that the DSI may vary with the brain areas in the output synapses of FSi.

Although we observed marked differences in the uIPSCs and their short-term modulation in the synapses made by STi and other INs, the inhibitory transmission appeared highly reliable in all synapses irrespective of the subtype identity of the presynaptic INs. Of particular note was that in the STi-PN synapses, any single firing of STi was found to unexceptionally evoke uIPSCs in PNs. To our knowledge, such highly reliable synaptic transmission was very seldom reported for other IN subtypes or in other brain areas [44]. Thus, despite STi only constituting a relatively small fraction of the IN population, the reliable, fast and robust inhibition together with its low capacity of short term modulation may confer a spectacular role onto STi in gating the activity of LA and shaping its output to the downstream basal and central amygdala. Since the excitatory synaptic inputs also vary among IN subtypes and have considerable impact on the functional involvement of different INs in the local circuit [15], [45], to gain more insight into the engagement of STi in LA circuit, it is necessary to identify the potential differences in the excitation pattern received by STi and other INs.

## Supporting Information

Figure S1 Comparison of the uIPSCs evoked in synapses made by distinct INs in basal amygdala. Comparisons of the amplitude (A), charge transfer (B), onset latency (C), 10–90% rise time (D) and decay time constant (E) of the uIPSCs in synapses made by STi (n = 4), FSi (n = 6) and LSi (n = 5) onto PNs. *p<0.05, **p<0.01, ***p<0.0001.
(TIF)
