# Correction: Stuttering Interneurons Generate Fast and Robust Inhibition onto Projection Neurons with Low Capacity of Short Term Modulation in Mouse Lateral Amygdala

**DOI:** 10.1371/annotation/3bc30ead-f84a-434a-8314-4aa1c9fcdcc0

**Published:** 2013-06-19

**Authors:** Chen Song, Xiao-Bin Xu, Ye He, Zhi-Peng Liu, Min Wang, Xin Zhang, Bao-Ming Li, Bing-Xing Pan

In the Funding section, one of the grant numbers from the funding body National Natural Science Foundation of China is incorrect. The correct numbers of the grants from this funding body are: 81071096, 31160208. 

